# Early Gastric Cancer Detected During 15‐year Surveillance After Ménétrier Disease Remission and Successfully Treated With Endoscopic Submucosal Dissection: A Case Report

**DOI:** 10.1002/deo2.70389

**Published:** 2026-08-26

**Authors:** Takaaki Shamoto, Hideko Yamamoto, Takehito Naito, Masahiro Yamada, Hirotaka Suzuki, Emiko Hida, Takafumi Yamamoto, Yoshifumi Arai, Hiroshi Matsubara

**Affiliations:** ^1^ Department of Gastroenterology Toyohashi Municipal Hospital Aichi Japan; ^2^ Department of Pathology Toyohashi Municipal Hospital Aichi Japan

**Keywords:** endoscopic submucosal dissection, endoscopic surveillance, gastric cancer, Ménétrier disease, protein‐losing enteropathy

## Abstract

Ménétrier disease (MD) was diagnosed based on hypoalbuminemia and giant gastric folds extending from the body to the fundus in a woman in her 70s. A serum anti‐*Helicobacter pylori* antibody test and urea breath test yielded negative results. Remission was achieved with steroid therapy, and she was relapse‐free with the use of a proton pump inhibitor. Annual esophagogastroduodenoscopy (EGD) was continued for gastric cancer surveillance. Fifteen years after the diagnosis of MD, a type 0‐I well‐differentiated tubular adenocarcinoma was detected on the posterior wall of the upper gastric body. EGD, computed tomography, and endoscopic ultrasonography revealed no findings suggestive of MD recurrence, and the lesion was considered intramucosal. Endoscopic submucosal dissection resulted in curative en bloc resection. Histopathology revealed no evidence of active MD. The patient remained free of MD relapse and gastric cancer recurrence. MD is characterized by giant gastric folds and protein‐losing gastroenteropathy, and it is considered a risk factor for gastric cancer. However, reports of gastric cancer diagnosed at an early stage and treated endoscopically during long‐term follow‐up after clinical remission induced by medical therapy, as in the present case, are rare. This case highlights the importance of continued endoscopic surveillance for gastric carcinogenesis even after long‐term clinical remission of MD.

## 1 Introduction

Ménétrier disease (MD) is a rare hypertrophic gastropathy characterized by giant folds in the gastric body and fundus, foveolar hyperplasia, and protein‐losing enteropathy (PLE) [1]. MD is associated with *Helicobacter pylori* infection, and it is considered a risk factor for gastric cancer [2]. However, an early diagnosis of gastric cancer coexisting with MD is often difficult because the giant folds can obscure neoplastic lesions. Therefore, surgery has been performed for symptom control or because of concerns regarding malignancy. However, some cancers have been identified only after gastrectomy. Reports of early gastric cancer diagnosed and treated endoscopically during long‐term follow‐up after medically induced remission of MD are rare. We report an *H. pylori*‐negative case of MD in which early gastric cancer was detected 15 years after remission and treated successfully with endoscopic submucosal dissection (ESD).

## 2 Case Report

**FIGURE 1:**
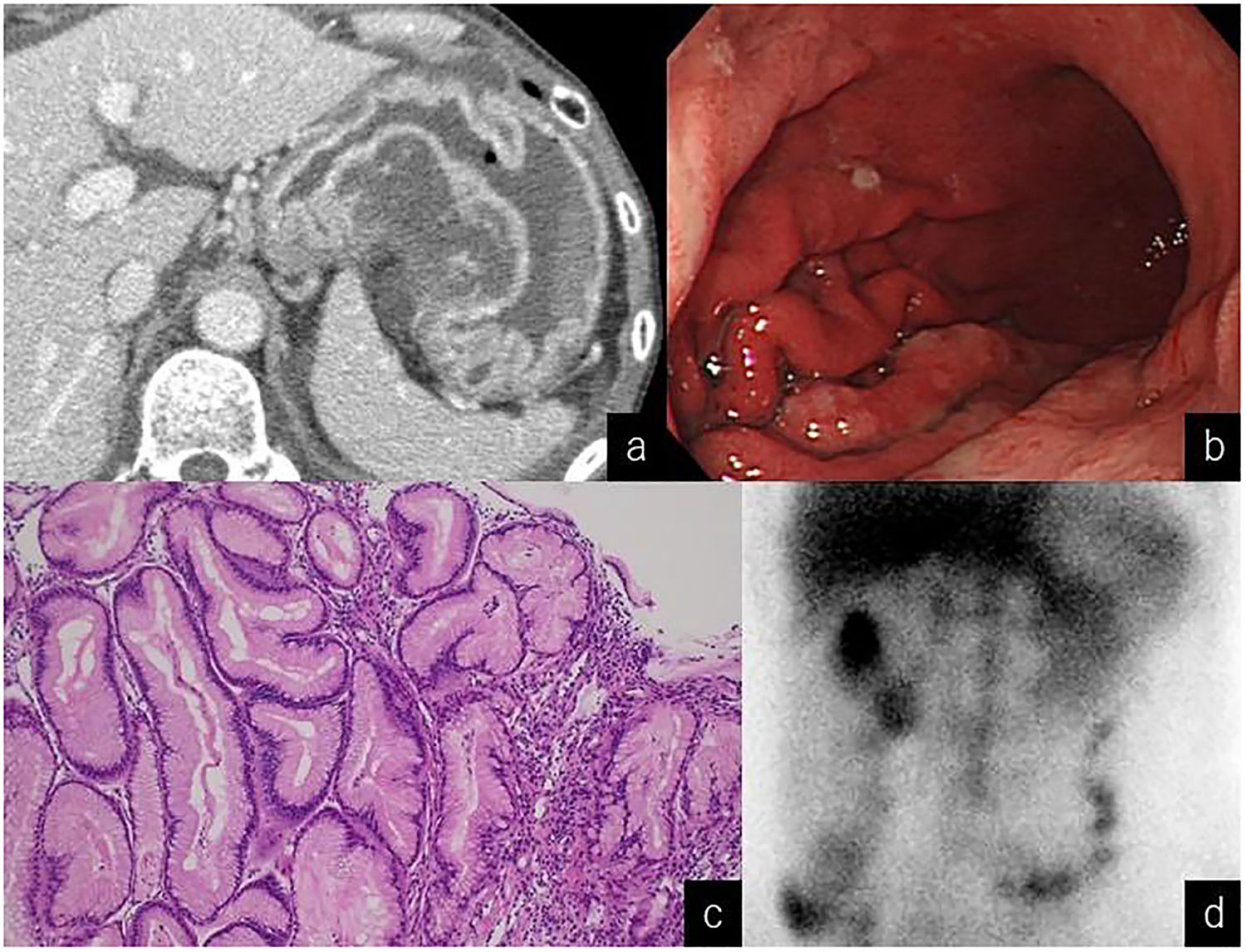
(a, b) Contrast‐enhanced computed tomography and esophagogastroduodenoscopy reveal marked gastric wall thickening extending from the gastric body to the fundus. (c) Histopathology (hematoxylin and eosin stain, ×100). Gastric mucosal biopsy findings indicate foveolar epithelial hyperplasia. (d) Protein‐losing scintigraphy confirms protein leakage from the stomach.

A woman in her 70s presented on February 2010 with edema of the upper and lower extremities, diarrhea, and epigastric discomfort. Laboratory testing revealed hypoproteinemia and hypoalbuminemia, with a total protein level of 3.5 g/dL and an albumin level of 2.1 g/dL. Computed tomography demonstrated marked gastric wall thickening with massive pleural effusion and ascites (Figure 1a). EGD revealed giant gastric folds extending from the body to the fornix (Figure 1b). Biopsy specimens obtained from these lesions revealed foveolar hyperplasia (Figure 1c). Protein leakage scintigraphy demonstrated protein loss from the stomach (Figure 1d); therefore, PLE associated with MD was diagnosed.

**FIGURE 2:**
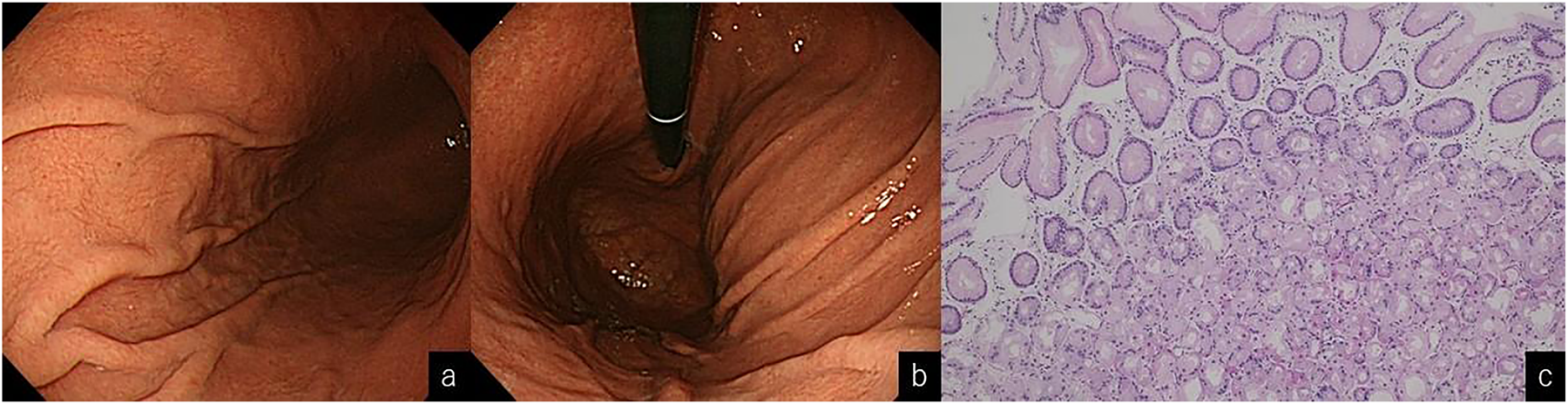
(a, b) Esophagogastroduodenoscopy demonstrates resolution of gastric wall thickening. (c) Histopathology (hematoxylin and eosin stain, ×100). Gastric mucosal biopsy findings indicate foveolar epithelial hyperplasia improvement.

The serum anti–*H. pylori* immunoglobulin G antibody test and a urea breath test yielded values of 7.0 U/mL (cutoff, <10.0 U/mL) and 0.9‰ (cutoff, 0%–2.4‰), respectively, both within the negative range. Furthermore, *H. pylori* infection was not identified in biopsy specimens. Cytomegalovirus infection was not detected. Steroid therapy was initiated on March 2010. Hypoalbuminemia and other symptoms improved promptly, and regression of the giant folds was observed endoscopically (Figure 2a). A follow‐up biopsy revealed improvement in foveolar hyperplasia (Figure 2b). Steroids were gradually tapered and discontinued, and remission continued with a proton pump inhibitor (lansoprazole, 10 mg/day).

**FIGURE 3:**
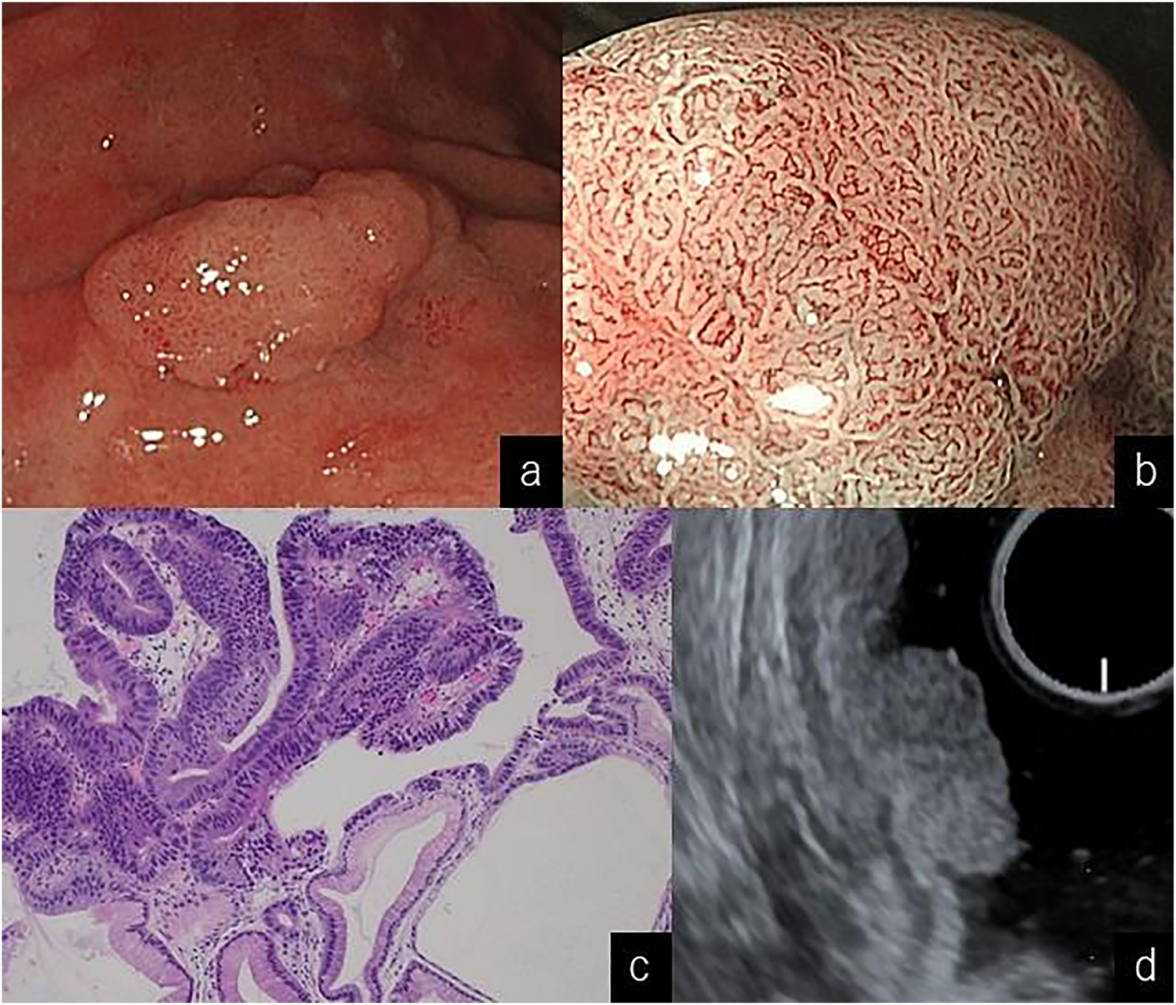
(a) A type 0‐I protruding lesion is identified on the posterior wall of the upper gastric body. (b) Magnifying narrow‐band imaging reveals irregular microvascular and microsurface patterns with a clear demarcation line. (c) Histopathology (hematoxylin and eosin stain, ×100). Biopsy findings confirm well‐differentiated tubular adenocarcinoma. (d) Endoscopic ultrasonography demonstrates thickening confined to the first layer without invasion of the second layer. The surrounding mucosa appears normal.

**FIGURE 4:**
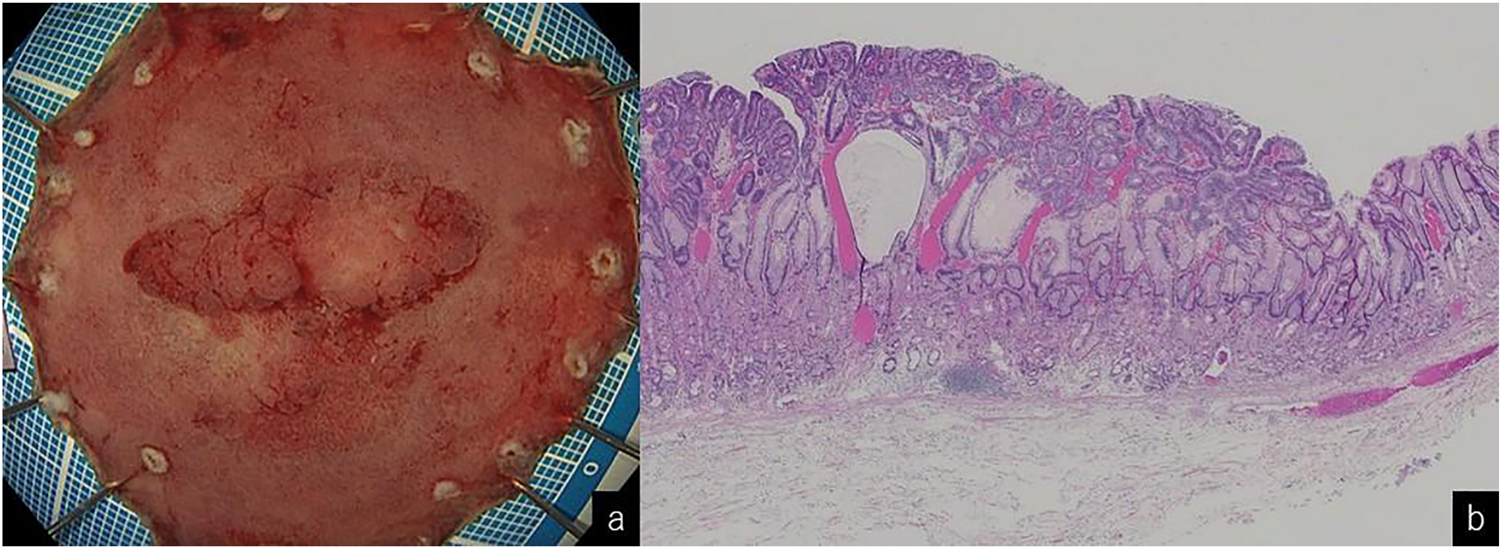
(a) En bloc resection of the lesion using endoscopic submucosal dissection. (b) Histopathology (hematoxylin and eosin stain, ×25). The tumor is diagnosed as a moderately differentiated tubular adenocarcinoma (tub2) with the following characteristics: 22 mm × 18 mm, pT1a (M), Ly0, V0, HM0, VM0, and UL0. Foveolar epithelial hyperplasia suggestive of active Ménétrier disease is not observed in the background mucosa.

Thereafter, EGD was performed annually for gastric cancer surveillance. In June 2010+15, EGD revealed no findings suggestive of recurrent MD; however, a protruding type 0‐I lesion was identified on the posterior wall of the upper gastric body (Figure 3a). Magnifying endoscopy with narrow‐band imaging demonstrated a demarcated area with irregular microvascular and microsurface patterns (Figure 3b). A biopsy revealed well‐differentiated tubular adenocarcinoma (Figure 3c). Endoscopic ultrasonography showed thickening limited to the first layer, with no obvious invasion of the second layer (Figure 3d). Additionally, no thickened folds suggestive of active MD were observed in the surrounding mucosa. Therefore, the lesion was diagnosed as an intramucosal cancer. ESD performed on August 2010+15 resulted in en bloc resection (Figure 4a). Histopathology confirmed carcinoma confined to the mucosa without lymphovascular invasion and negative horizontal and vertical margins, indicating curative resection (tub2, pT1a[M], Ly0, V0, HM0, VM0, and UL0; Figure 4b). Furthermore, no histopathological findings suggested active MD. The patient remained free of MD relapse and gastric cancer recurrence after ESD.

## 3 Discussion

MD often causes hypoalbuminemia, edema, and weight loss because of PLE. The diagnosis is based on a combination of clinical, endoscopic, and histopathological findings [3]. For patients with MD, medical therapy primarily aims to improve PLE and related symptoms. *H. pylori* eradication is recommended for positive cases; however, proton pump inhibitors and steroids may improve symptoms and mucosal abnormalities [4, 5]. In the present case, testing revealed *H. pylori*‐negative results, and steroid therapy induced both clinical and endoscopic remission.

Immunohistochemical staining was performed to evaluate the mucin phenotype in our case. The tumor cells were negative for MUC1 and MUC2, positive for MUC5AC and MUC6, and focally positive for CD10 (Figures S1–S5). Based on these expression patterns, the tumor's mucin phenotype was classified as gastric [6]. Additionally, the background mucosa exhibited no intestinal metaplasia, and gastric foveolar epithelium—although without marked hyperplasia—was observed immediately beneath the cancerous lesion. MD is primarily characterized by gastric foveolar hyperplasia, and it is well established that the activation of the TGF‐α/EGFR pathway drives a continuous proliferative state of the gastric‐type epithelium [7]. Therefore, the gastric mucin phenotype observed in this case may suggest a biological continuity with the gastric foveolar epithelial lineage that undergoes hyperplasia in MD. Furthermore, the tumor was located immediately above the gastric foveolar epithelium, and the background mucosa completely lacked intestinal metaplasia. Fukushi et al. proposed that a gastric adenocarcinoma with a gastric mucin phenotype arising in MD with foveolar hyperplasia may have developed through the hyperplasia–dysplasia–carcinoma sequence rather than through the conventional Correa cascade involving intestinal metaplasia [8]. Although other potential confounding factors, such as long‐term proton pump inhibitor use, should also be considered, the present case suggests that a microenvironment unique to MD may have contributed to the development of this tumor.

A 2014 review reported that more than 50 cases of gastric cancer associated with MD have been described worldwide and suggested that MD should be considered a premalignant condition [9]. Additionally, a case‐control study showed that gastric cancer developed in 8.9% of patients with MD within 10 years, which was significantly higher than the rate of 3.7% observed in the control group [2]. These reports indicate that MD should be considered a high‐risk factor for gastric cancer, and that regular endoscopic surveillance is necessary. Nevertheless, early diagnosis of gastric cancer in MD is difficult because giant folds may mask neoplastic lesions. Historically, many patients have undergone surgery for symptom control or suspected malignancy, and coexisting cancer was sometimes recognized only after resection. However, when the nutritional status is impaired by PLE, the invasiveness of surgery can impose a substantial burden on the patient and lead to deterioration in the quality of life (QOL). Therefore, if gastric cancer can be diagnosed at an early stage, then endoscopic treatment may preserve gastric function and improve QOL. Nevertheless, because surgery has been performed for many cases of MD, including for symptom control, reports of the diagnosis and endoscopic treatment of early‐stage gastric cancer arising in MD are rare.

A PubMed search conducted on April 12, 2026 using the keywords (“Ménétrier's disease” [All Fields] OR “Ménétrier disease” [All Fields] OR “Menetrier disease” [All Fields]) AND (“early gastric cancer” [All Fields] OR “early gastric carcinoma” [All Fields]), identified only two cases in which early gastric cancer with MD was confirmed by endoscopy prior to any therapeutic intervention and managed endoscopically. One case was treated with endoscopic mucosal resection, and another with ESD [8, 10]. In both cases, treatment was performed for early gastric cancer detected at the time of the initial diagnosis of MD, suggesting that endoscopic treatment may contribute to improved QOL. In contrast, our case is a rare example of gastric cancer arising during long‐term follow‐up after clinical remission of MD that was diagnosed at an early stage and treated endoscopically. During routine EGD surveillance, a retrospective review of prior endoscopic images revealed that the lesion could have been identified 3 years before diagnosis. At that time, the lesion appeared as a minute, mildly erythematous, flat lesion, and the elevated component remained subtle on endoscopy (Figure S6). Because the lesion was not considered suspicious, no biopsy was performed, precluding histopathological diagnosis at that time. Subsequently, the lesion gradually enlarged, and its demarcation from the surrounding mucosa became more distinct, allowing endoscopic recognition and histopathological diagnosis at the next EGD examination in 2010+15. Therefore, during long‐term management of MD, endoscopic surveillance aimed at detecting early gastric cancer is important after clinical remission to improve survival and maintain QOL. We believe that this case supports the importance of such surveillance.

## Author Contributions

**Takaaki Shamoto**: conceptualization, methodology, data curation, investigation, visualization, project administration, writing – original draft, and writing – review & editing. **Hideko Yamamoto**, **Takehito Naito**, **Masahiro Yamada**, **Hirotaka Suzuki**, **Emiko Hida**, **Takafumi Yamamoto**, and **Hiroshi Matsubara**: writing the review and editing. **Yoshifumi Arai**: evaluation of pathological specimens. All authors reviewed and approved the final version of the manuscript. **Takaaki Shamoto** is the article guarantor.

## Funding

The authors have nothing to report.

## Ethics Statement

Written informed consent has been obtained from the patient to publish this paper.

## Conflicts of Interest

The authors declare no conflicts of interest.

## Supporting information




**FIGURE S1**: Immunohistochemical staining for MUC1 (×100). The tumor cells were negative for MUC1.

**FIGURE S2**: Immunohistochemical staining for MUC2 (×100). The tumor cells were negative for MUC2.

**FIGURE S3**: Immunohistochemical staining for MUC5AC (×100). The tumor cells were positive for MUC5AC.

**FIGURE S4**: Immunohistochemical staining for MUC6 (×100). The tumor cells were positive for MUC6.

**FIGURE S5**: Immunohistochemical staining for CD10 (×100). The tumor cells were focally positive for CD10.

**FIGURE S6**: Retrospective review of the previous endoscopic images showed that the lesion was already present in the upper gastric body as a very small, slightly reddish, flat lesion in 20XX+12.

**FILE S1**: deo270389‐sup‐0007‐FiguresCaption.docx

## Data Availability

The data underlying this article are available in the article.
