# Supplementary material for: Early Gastric Cancer Detected During 15‐year Surveillance After Ménétrier Disease Remission and Successfully Treated With Endoscopic Submucosal Dissection: A Case Report
Source: DEN Open. 2026 Aug 26;7(1):e70389. doi: 10.1002/deo2.70389 (PMC13510617; doi:10.1002/deo2.70389)
Supplement: Supplementary file 1 [file DEO2-7-e70389-s007.docx]

**Supplementary Figure Legends**

Supplementary Figure1: Immunohistochemical staining for MUC1 (×100). The tumor cells were negative for MUC1.

Supplementary Figure2: Immunohistochemical staining for MUC2 (×100). The tumor cells were negative for MUC2.

Supplementary Figure3: Immunohistochemical staining for MUC5AC (×100). The tumor cells were positive for MUC5AC.

Supplementary Figure4: Immunohistochemical staining for MUC6 (×100). The tumor cells were positive for MUC6.

Supplementary Figure5: Immunohistochemical staining for CD10 (×100). The tumor cells were focally positive for CD10.

Supplementary Figure6: Retrospective review of the previous endoscopic images showed that the lesion was already present in the upper gastric body as a very small, slightly reddish, flat lesion in 20XX+12.
